# Modelling mitochondrial diseases in neurons *In Vitro*: A systematic review

**DOI:** 10.1177/22143602241307198

**Published:** 2025-06-19

**Authors:** Mariana Zarate-Mendez, Nihal A Basha, Oliver Podmanicky, Natalia Malig, Denisa Hathazi, Rita Horvath

**Affiliations:** 1 Department of Clinical Neurosciences, University of Cambridge, Cambridge, UK; 2 School of Clinical Medicine, University of Cambridge, Cambridge, UK

**Keywords:** induced pluripotent stem cells, primary mitochondrial diseases, neuronal models

## Abstract

Mitochondrial diseases, characterized by disruptions in cellular energy production, manifest diverse clinical phenotypes despite a shared molecular aetiology. Of note is the frequent involvement of the brain in these pathologies. Given the inherent challenges associated with accessing human tissue and the limitations of mouse models, especially concerning mitochondrial DNA (mtDNA), in vitro modelling is crucial in elucidating brain-related manifestations of mitochondrial diseases.

In this review we recapitulate the current available in vitro models used to study neuronal cell types and advance our understanding of mitochondrial brain disease. This inquiry is especially pertinent considering the scarcity of suitable animal models, necessitating reliance on in vitro models to elucidate underlying molecular mechanisms. We found fifty papers modelling neuronal mechanisms of mitochondrial diseases in-vitro. While there was an even split between nuclear and mtDNA mutations, MELAS was the most commonly modelled syndrome. The emerging technologies in the stem cell field have revolutionized our approach to investigate cellular specificity in mitochondrial diseases, and we found a clear shift from neuroblastoma cell lines to iPSC-derived models. Interestingly, most of these studies reported impaired neuronal differentiation in mutant cells independent of the syndrome being modelled. The generation of appropriate in vitro models and subsequent mechanistic insights will be central for the development of novel therapeutic avenues in the mitochondrial field.

## Introduction

Mitochondrial diseases are clinically very heterogeneous conditions caused by defective oxidative phosphorylation.^1^ Neuronal cells are metabolically active and rely largely on ATP generated through oxidative phosphorylation. Therefore, debilitating neurologic manifestations are common in mitochondrial diseases.^2^ Understanding the molecular mechanisms leading to neurological symptoms in these disorders is a promising avenue to develop better treatments. However, research efforts have been hampered in the past by the difficulty in generating animal models and in vitro cellular models for mitochondrial diseases. With recent advances in the field, there are more possibilities to model neurological mechanisms, and we decided to review the information available for modelling mitochondrial dysfunction *in vitro* in neurons affected by primary mitochondrial diseases.

Defining the term ‘primary mitochondrial disease’ (PMD) is a contentious issue in the literature Rahman et al., definePMDs as “genetic disorders leading either to oxidative phosphorylation dysfunction or other disturbances of mitochondrial structure and function”. This definition was used in this systematic review. The mutations underlying PMDs may arise in mitochondrial DNA (mtDNA) or nuclear DNA (nDNA). The mtDNA is a small 16.5 kb circular molecule that consists of 37 genes which encode 13 respiratory chain protein subunits, 22 transfer RNAs (tRNAs) and 2 ribosomal RNAs (rRNAs), required for mitochondrial protein synthesis.^3^^,^^4^ However, most of the over 1500 proteins required for mitochondrial function are encoded by the nDNA, translated in the cytoplasm, and then imported into the mitochondria.^5^ nDNA mutations underlying PMDs occur in genes with roles in respiratory chain complexes or their assembly factors, components of the mitochondrial protein synthesis machinery, mtDNA maintenance, fusion/fission, or metabolism of cofactors and vitamins.^6,7^

*In vivo* animal models of neurologic manifestations of PMDs have limitations to their utility. For example, murine models are among the most commonly used in neuroscience research^8^ but have failed to recapitulate many neurological pathologies seen in patients.^9^ Indeed, while there has been a few successful animal models able to recapitulate some clinical features of mitochondrial disease, such as the mouse model of Leigh syndrome,^10^ most attempts have resulted in embryonic lethality or lack relevant phenotypes. For instance, a large effort to develop a mouse model for disorders related to Polγ mutations have consistently shown that knockout of either the catalytic or the accessory subunit is embryonic lethal while heterozygous mice are undiscernible from controls.^11,12^ Furthermore, the *Polg^D257A^* mutator mouse accumulates mtDNA mutations but lacks the common phenotypes observed in patients.^13^ Additional attempts to develop mouse models for nuclear genes causing mitochondrial disease have also been limited to very mild phenotypes, such as the TWINKLE deletor^14^ and the SURF1 knockout mice.^15^ Moreover, since mtDNA is quite resilient to genetic manipulation and modifications, our ability to further create appropriate rodent models that can be used to study PMDs caused by mutations in mtDNA is limited.

While *in silico* modelling presents a rapid and inexpensive method for investigating variants underlying mitochondrial diseases,^16^ a range of modelling techniques are required for the comprehensive study of PMDs. *In vitro* models are therefore an important avenue for studying the neurologic manifestations of PMDs, the disease mechanisms and potential therapeutic approaches. Current *in vitro* models range from cybrids, typically generated by fusing enucleated cells with those with depleted mtDNA,^17^ to human embryonic stem cells (hESCs) and induced pluripotent stem cells (iPSCs) differentiated into disease-relevant cell types. Furthermore, due to advances in bioengineering, cultures are no longer restricted to two dimensions. New types of 3-dimensional cultures, such as spheroids, organoids, and microfluidic ‘organ-on-a-chip’ systems, can now be generated.^18^ Other developments, such as reprogramming somatic cells into induced neuronal progenitors without using iPSCs as an intermediary^19^ have further broadened the repertoire of *in vitro* models. It is important to emphasize that mitochondrial diseases are tissue specific and thus the phenotypes observed in disease models are in part dictated by the cell type used. For example, in mitochondrial encephalomyopathy, lactic acidosis, and stroke-like episodes (MELAS) syndrome, distinct changes were observed in fibroblasts, iPSCs, and neurons,^20^ highlighting the importance of using cell types relevant to the pathology.

Given the heterogeneity of mitochondrial diseases, the variety of associated syndromes and the large range of disease-causing mutations, it is challenging to get a clear overview of all available studies modelling PMDs in neurons. Moreover, the differential effect of each mutation in neuronal pathology further hinders the effort to draw conclusions about possible convergent disease mechanisms. To obtain a comprehensive summary of existing *in vitro* models of PMDs using relevant neuronal cell types we performed a systematic review. The aim of our study is to provide an overview of the available tools, the key findings from these models, and to evaluate the advantages and disadvantages of these novel technologies in modelling different manifestations of PMD.

## Methods

### Eligibility criteria

The aim was to identify all available *in vitro* models of human neurons, glial cells, and their progenitors reported to study PMDs in humans. As there have been recent reviews on *in vitro* models of mitochondrial optic neuropathies, such as autosomal dominant optic atrophy (ADOA) and Leber hereditary optic neuropathy (LHON),^21,22^ we excluded papers on retinal ganglion cells (RGCs), though other neuronal cellular models of these conditions are part of this review. The complete inclusion and exclusion criteria are outlined in *Table 1*. Records/reports were required to meet all inclusion criteria and not meet any criteria for exclusion.

**Table 1.** Inclusion and exclusion criteria for title and abstract and full text screening.

**Inclusion Criteria**
Peer reviewed Primary research Uses at least 1 *in vitro* model consisting of human neurons/neuronal precursors, neuroblastoma cells, glia/glial precursors, or inner ear hair cells to study a named PMD or a mutation strongly linked to a PMD that affects oxidative phosphorylation or mitochondrial structure or function, as the principal pathology
**Exclusion Criteria**
Not in the English language Uses only models consisting of retinal tissue, RGCs, or optic nerve tissue Retracted

### Information sources and search strategy

PubMed was searched on the 17^th^ of January 2023 and an outline of the search components and structure is provided below.

Search components:

1. Named mitochondrial syndromes in the form of free words, connected with ‘OR’
2. Named mitochondrial syndromes in the form of MeSH terms, connected with ‘OR’
3. Genes in which mutations are strongly linked to PMDs in the form of free words, connected with ‘OR’
4. Synonyms for PMD in the form of free words, connected with ‘OR’
5. Terms about *in vitro* modelling in the form of MeSH terms, connected with ‘OR’
6. Terms about *in vitro* modelling in the form of free words, connected with ‘OR’
7. Terms relating to irrelevant *in vitro* techniques or neoplasms in the form of free words and MeSH terms, connected with ‘OR’

Search structure: (((1 OR 2) OR (3) OR (4)) AND (5 OR 6)) NOT (7)

No filters were used. All records were uploaded to Zotero and Rayyan. The Rayyan function to automatically detect duplicates was used, these were manually verified and removed.

### Selection process

First, 2 blinded independent reviewers (NB and MZ-M) conducted title and abstract screening of 102 records. The percentage agreement was 100%. Therefore, the inter-rater reliability was considered sufficient to justify the remaining records being divided between NB and MZ-M for title and abstract and full text screening, rather than dual screening. The Zotero function to automatically detect retracted papers was used; these were manually verified and removed during title and abstract screening. Records/reports causing uncertainty were discussed at a meeting with NB, MZ-M and an expert reviewer (RH), to reach a resolution.

### Data collection process and data items

Papers included in the review were divided between 2 independent reviewers (NB and MZ-M). Data was collected in the following categories: phenotype, gene, mutation, model, key findings, and reference. Grouping of the included papers was based on the phenotype modelled*.* Due to the nature of the data collected and its intended use, it was decided that risk of bias assessment was not required.

## Results

**Figure 1.**
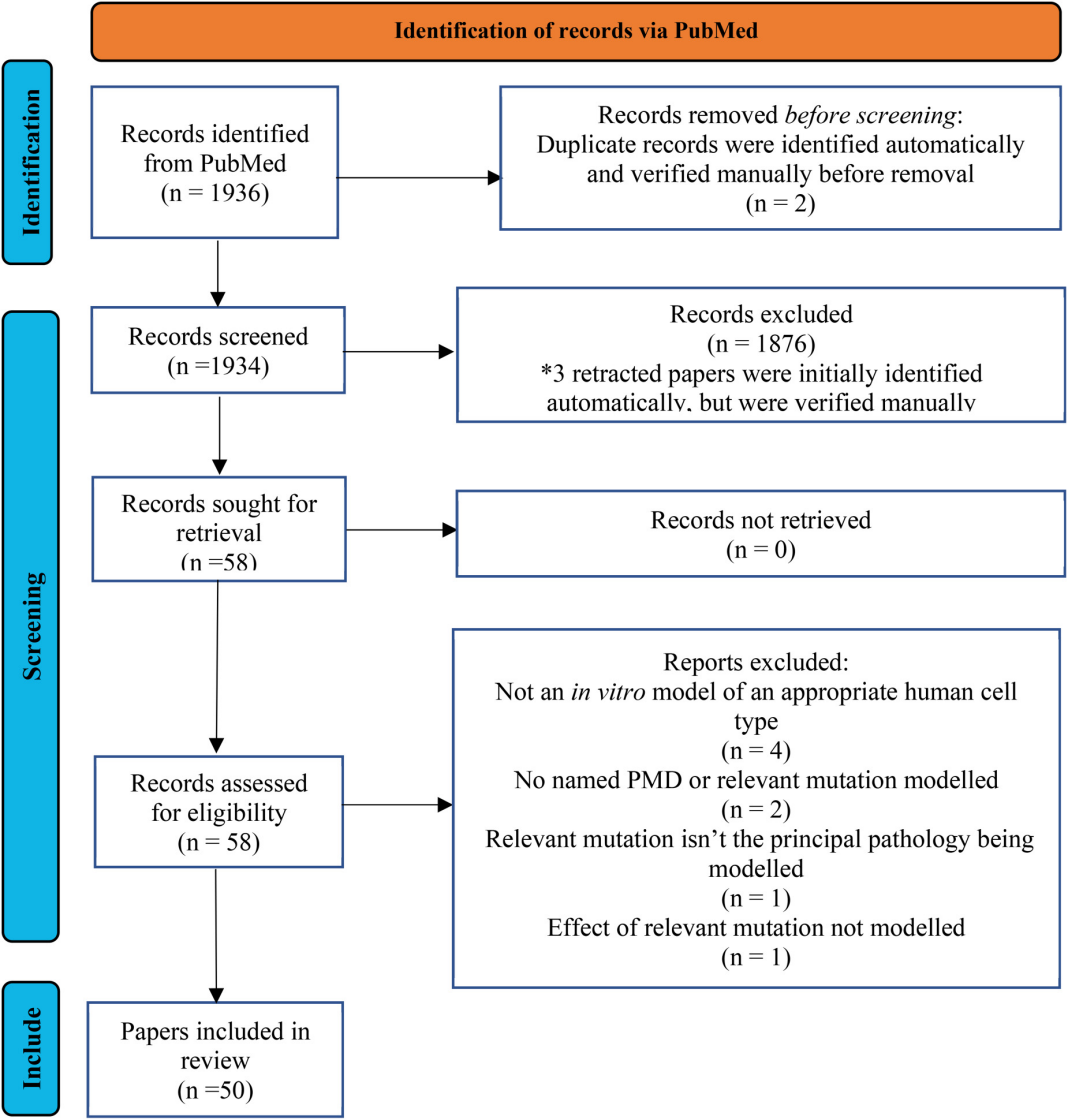
PRISMA flow diagram of the selection process.

**Figure 2.**
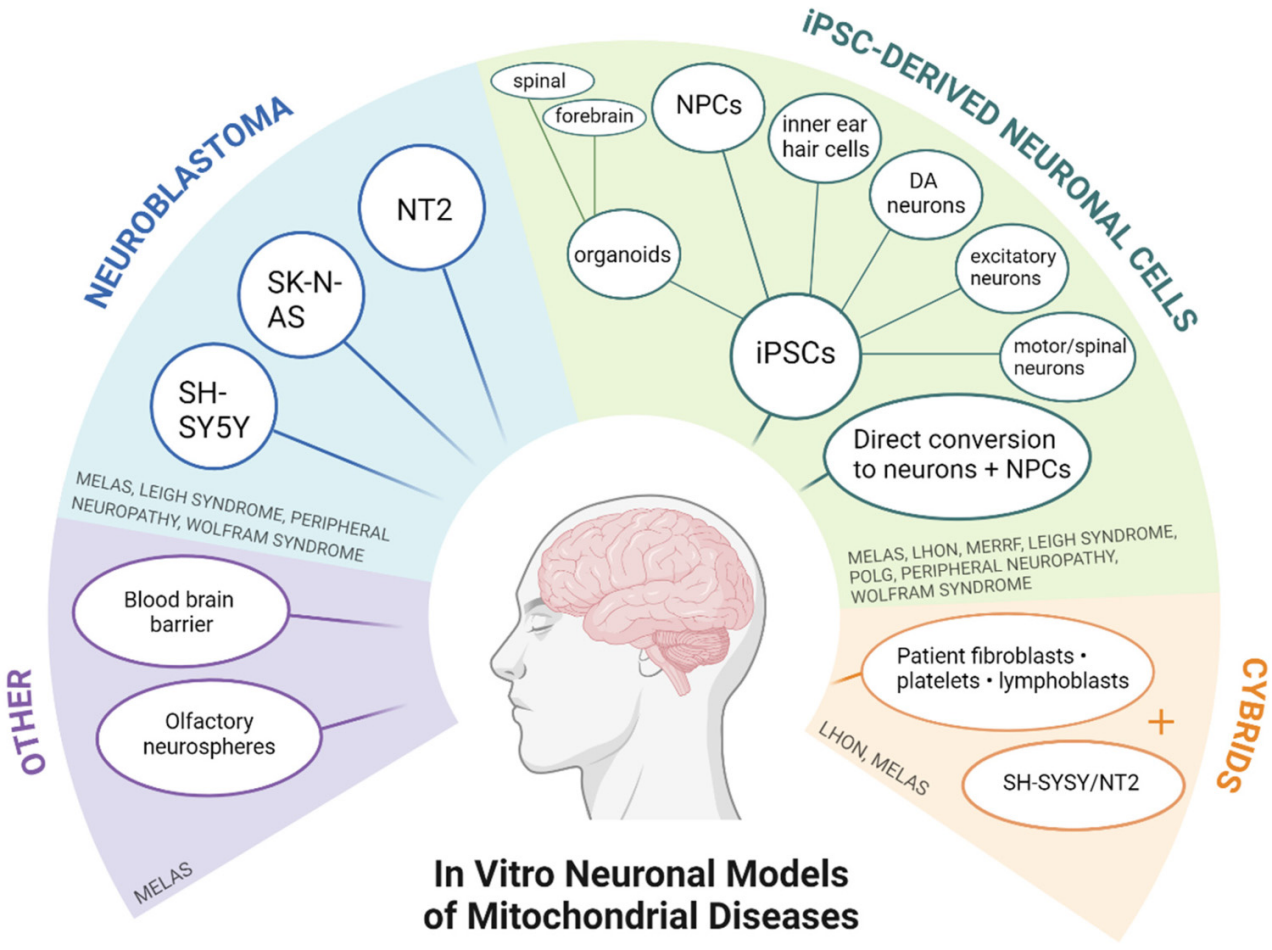
Summary diagram of published neuronal models for primary mitochondrial diseases. Created with BioRender.com.

In total 1936 records were returned by the search. Two duplicates were removed before screening, 1876 were excluded through title and abstract screening and 8 more through full text screening, leaving 50 papers to be included in the review. A Preferred Reporting Items for Systematic Reviews and Meta-Analyses (PRISMA) flow diagram of the selection process is provided in *Figure 1*. Neuronal models most represented on these papers included neuroblastoma cells, cybrids and iPSCs differentiated into various neuronal cell types, with MELAS being the most frequently modelled syndrome. A summary of all *in vitro* models and the respective mitochondrial diseases can be found in *Figure 2.*

### Modelling diseases arising from nuclear DNA mutations

We found 27 papers modelling PMD caused by mutations in nDNA, which are summarised in *Table 2*. Mutations underlying Charcot-Marie-Tooth (CMT) have been extensively studied in patient-derived neurons to understand the mechanisms of neuropathy. We identified six papers studying CMT; four modelled the disease in iPSC-derived motor neurons with defects in either MFN2,^32,34,35^ or GDAP1,^37^ one group used SH-SY5Y neuroblastoma cells carrying GDAP1 mutations^36^ and another modelled GARS1 mutations in induced NPCs.^33^ Fang et al.^48^ also investigated MFN2 in human iPSC-derived cortical neurons in a wider context of mitochondrial dynamics and neuronal development. Altered mitochondrial dynamics, increased mitophagy, and changes to calcium homeostasis were consistently identified in these models.

**Table 2.** Papers modelling diseases arising from nuclear DNA mutations abbreviations: dopaminergic (DA), Complex I, IV,V (CI, CIV,CV).

Reference	Gene and Mutation	Model	Key Findings
**POLG-related disorders**			
^23^	c.1681G > A (p.A467 T) c.2243G > C (p.W748S) c.3800insGACT (p.L1173fsX)	Patient-derived iPSCs differentiated to excitatory cortical neurons	Decreased mitochondrial content, mtDNA depletion, abnormal mitochondrial function and structure, and morphological alterations. Overexpression of PINK1 rescued neurite complexity and ATP/ADT ratio.
^24^	c.2243G > C (p.W748S) c.1399G > A (p.A467 T)	Patient-derived iPSCs differentiated to DA neurons	Decreased mitochondrial membrane potential, CI, and mtDNA; increased oxidative stress. N-acetylcysteine rescued mitochondrial dysfunction
^25^	c.2243G > C (p.W748S) c.1399G > A (p.A467 T)	Patient-derived iPSCs differentiated to NPCs and DA neurons	Impaired ATP production, loss of mtDNA, increased oxidative stress, and loss of CI, disturbed NAD + metabolism &mitophagy in NSCs/DA neurons.
^26^	c.2243G > C (p.W748S) c.1399G > A (p.A467 T)	Patient-derived iPSCs differentiated to DA neurons and astrocytes	Abnormal mitochondrial remodelling; impaired mtDNA copy number, CI, NAD + metabolism; mitochondrial membrane potential and ATP production.
^27^	c.2243G > C (p.W748S) c.1399G > A (p.A467 T)	Patient-derived iPSCs differentiated to NPCs and astrocytes	Nicotinamide riboside and metformin rescued a mitophagy defect in astrocytes via upregulation of SIRT1/AMPK, and downregulation of mTOR.
**Leigh Syndrome**			
^28^	NDUSF4 knockout	iPSC differentiated to excitatory cortical neurons	Increased acetylation of p53 and apoptosis, which was attenuated by nicotinamide riboside treatment.
^29^	PDHA1:c.79delC (p.R27 fs) DLD: c.100A > G (p.T34A) MT-ATP6: m.8993T > G (p.L156R)	Patient-derived iPSCs differentiated to cerebral organoids	Mutations impaired corticogenesis, neural epithelial bud formation, mitochondrial morphology, cortical architecture, and caused astrocytosis due to defective metabolic switch from glycolysis to OXPHOS.
^30^	SURF1: c.530 T > G (p.V177G) /c.769G > A (p.G180R); NDUSF4: c.462delA(p.K154 fs) /c.316C > T(p.R106*)	Patient-derived iPSCs differentiated to NPCs, DA neurons and 3D cerebral organoids	SURF1 mutations disrupted neuronal maturation. NDUFS4 mutations led to similar defects in early neuronal morphogenesis suggesting a general pathogenetic mechanism of Leigh syndrome.
^31^	LRPPRC knockdown	SH-SY5Y	LRPPRC knockdown led to loss of CIV subunits and mitochondrial hyperfusion.
**Peripheral neuropathy**			
^32^	MFN2 knockdown	hESCs differentiated to spinal motor neurons	Impaired anterograde and retrograde mitochondrial transport in MFN2 knockdown motor neurons, fragmentation, and decreased mitochondrial membrane potential.
^33^	GARS1:c.647A > G (p.H216R) c.1904C > T (p.S635L) c1787G > A (p.R596Q)	Direct conversion of patient fibroblasts to iNPCs	Recessive GARS1 mutations led to reduced respiratory chain complex subunits and fatty acid oxidation, mitochondrial calcium uptake and autophagy was altered in dominant mutants. Downregulation of GARS1 impaired protein levels of respiratory chain subunits.
^34^	MFN2:c.1188C > T (p.A383 V)	Patient-derived iPSCs differentiated to motor neurons	Altered mitochondrial distribution, reduced mitochondrial content, and enhanced mitophagy
^35^	MFN2:c.1090C > T (p.R364 W)	Patient-derived iPSCs differentiated to motor neurons	Hyperexcitability, altered sodium and calcium channel kinetics and mitochondrial trafficking
^36^	GDAP1:c.358C > T (p.R120 W) JPH1: c.638G.C (p.R213P)	SH-SY5Y	JPH1 is a modifier for GDAP1 and can modify GDAP1 mutations by regulating calcium homeostasis
^37^	GDAP1: c.581C > G (p.S194*)	Patient-derived iPSCs differentiated to motor neurons	GDAP1 loss caused cristae defects, altered distribution and thickness, decreased cell viability, lipid dysfunction, and oxidative stress
**Wolfram syndrome**			
^38^	WFS1: c.1230_1233delCTCT (p.V412fs*440); c.2171C > T (p.P724L);c.1060_1062delTTC(p.F354del); c.1676C > A (p.A559D)	iPSC derived glutamatergic cortical neurons	Mutant neurons exhibited early developmental alterations in neurite outgrowth, aberrant neurite formation; rescuing WFS1 expression restored normal neurite outgrowths; Valproic acid rescued aberrant neurite outgrowth
^39^	WFS1: c.937C > T (p.H313Y); c.376G > A (p.A126 T) ; c.1838G > A (p.W613X) ; c.599delT (p.L200fs286Stop); c.2254G > T (p.E752Stop); c.1943G > A (p.W648X); c.2084G > T (p.G695 V)	Patient-derived iPSCs differentiated to NPCs	Identified calpain 2 as a key player in cell death mechanisms associated with Wolfram syndrome. Dantrolene treatment prevented cell death in patient-derived NPCs.
^40^	WFS1 knockdown with siRNA	SK-N-AS, NT2	WFS1 interacts with V1A subunit of the V-ATPase, which might be relevant for pump assembly in ER.
**Other**			
^41^	OPA1: c.2873_2876delTTAG (p.V903Gfs*3)	hESC differentiated to NPCs and neurons; iPSC to NPCs	Impaired development of GABAergic neurons, increased oxidative stress, altered transcriptional circuitry in NPCs, reduced expression of FOXG1
^42^	CoQ10 competitively inhibited	SH-SY5Y	CoQ10 deficiency in SH-SY5Y cells impaired lysosomal acidification which was partially rescued with CoQ10 supplementation.
^43^	TIMM8A & TIMM8B knockouts	SH-SY5Y	TIMM8A was identified as a novel factor for CIV assembly
^44^	CHCHD10: c.176C > T (p.S59L)	Patient-derived iPSCs differentiated to motor neurons	Abnormal mitochondrial morphology. Mutant neurons significantly more sensitive to staurosporine or glutamate-induced apoptosis.
^45^	TYMP: c.866A > C (p.E289A)/ c.1231_1243del	Patient-derived iPSCs differentiated to cerebral organoids	Successfully recapitulated the absence of thymidine phosphorylase in the various neuronal cell types within the organoid.
^46^	PMPC: c.523C > T (p.R175C) ; c.601G > C (p.A201P); c.524G > A (p.R175H); c.530T > G (p.V177G); c.1265T > C(p.I422 T)	iPSCs and hESCs differentiated to NPCs	Impaired mitochondrial processing protease (MPP), which affected iron-sulfur cluster biogenesis in mutant NPCs.
^47^	ATAD3A: c.1064G > A (p.G355D)	Patient-derived iPSCs differentiated to neurons	Imbalanced mitochondrial dynamics; altered mitochondrial network and upregulated basal autophagy.
^48^	MFN2 knockdown	iPSCs differentiated to neurons	Impaired mitochondrial network, synapse formation, and neuronal differentiation
^49^	CoQ10 competitively inhibited	SH-SY5Y	Decreased mitochondrial respiratory chain enzyme activities, increased mitochondrial oxidative stress, and reversal of CV activity.

Five papers investigated POLG-related disorders using patient fibroblasts converted to iPSCs. Of these, three differentiated iPSCs into dopaminergic neurons (DA), a neuronal population that has been shown to be particularly vulnerable to degeneration in post-mortem studies of patients with POLG mutations.^24–26^ Other groups have also differentiated iPSCs into astrocytes, glutamatergic cortical neurons, and NPCs.^23,27^ POLG mutations led to mitochondrial dysfunction in all neuronal models including mtDNA depletion, decrease in complex I, loss of mitochondrial membrane potential, increased oxidative stress, lower ATP production, and abnormal mitophagy. Interestingly, mitochondrial abnormalities caused by POLG mutations did not disturb the differentiation of NSCs into astrocytes when compared to controls. In contrast, POLG mutations impaired the neuronal differentiation potential of NPCs as compared to controls.^26^ Of note, N-acetylcysteine treatment rescued POLG-induced mitochondrial dysfunction.^25^ Moreover, double treatment using nicotinamide riboside and metformin rescued abnormal mitophagy by upregulating the SRT1/AMPK pathway and downregulating mTOR.^27^

Five papers studied Leigh syndrome and related disorders using 3D cerebral organoids,^29,30^ iPSC-derived cortical neurons,^28^ SH-SY5Y neuroblastoma cells^31^ and neuroepithelial stem cells derived from a patient presenting with a Leigh-like phenotype.^46^ In the two studies using cerebral organoids, NDUFS4, SURF1 and PDHA1 mutations impaired corticogenesis and neuronal maturation by preventing the switch from glycolysis to OXPHOS during cortical development, which was accompanied by increased generation of astrocytes. Both viral-based SURF1 gene augmentation and pharmacological-based activation of mitochondrial biogenesis with bezafibrate restored healthy neuronal maturation by enabling the metabolic shift from glycolysis to OXPHOS.^30^ Abnormal mitochondrial dynamics were observed in NDUFS4-knockout cortical neurons,^28^ and in LRPPRC-knockdown SH-SY5Y cells.^31^ Moreover, Yoon et al.^28^ reported p53 hyperacetylation associated with neuronal excitotoxicity and apoptosis. Nicotinamide riboside treatment significantly decreased p53 acetylation and attenuated neuronal apoptosis.

Wolfram syndrome caused by WFS1 mutations was modelled in three papers. Two groups used patient-derived iPSCs differentiated into either cortical neurons^38^ orNPCs.^39^Gharanei et al.^40^ used siRNA to knockdown WFS1 in NT2 and SK-N-AS neuroblastoma cell lines. WFS1 mutations caused aberrant neurite formation during early neuronal development possibly due to altered expression of axon guidance genes.^38^ Lu et al.^39^ identified calpain 2 as a mediator of cell death underlying the pathomechanism of Wolfram syndrome. Amongst the pharmaceutical treatments, valproic acid rescued aberrant neurite outgrowth, and dantrolene prevented cell death by blocking calpain 2 activation and calcium release from the ER. Gharanei et al.^40^ showed that WFS1 is vital for proton pump assembly in the ER by its interaction with the V1A subunit of the H + -ATPase.

Disorders caused by CoQ10 biosynthesis defects belong to a potentially treatable subgroup of mitochondrial diseases. CoQ10 deficiency often leads to cerebellar ataxia and seizures, but its underlying mechanisms remain poorly understood. Two papers investigated CoQ10 deficiencies in the SH-SY5Y model using para-aminobenzoic acid (PABA) as an inhibitor of the CoQ10 biosynthetic pathway.^42,49^ Inhibiting CoQ10 in SH-SY5Y cells impaired activity of all mitochondrial respiratory complexes, and decreased ATP levels.^49^ In addition to its role as an essential electron carrier within the mitochondrial respiratory chain, Heaton et al.^42^ showed that CoQ10 inhibition impaired acidification of the lysosome which was partially restored by CoQ10 supplementation.

Caglayan et al.^41^ showed that OPA1 haploinsufficiency selectively impaired the development of GABAergic neurons but not glutamatergic neurons. In NPCs, OPA1 haploinsufficiency increased methylation of FOXG1 promoter and repressed the expression of FOXG1. On the other hand, Kang et al.^43^ used TIMM8A and TIMM8B knockout SH-SY5Y cells to model Mohr-Tranebjaerg syndrome. They identified a novel factor for complex IV assembly mediated through its transient interaction with other assembly factors, particularly the copper chaperone COX17. Depletion of TIMM8A led to oxidative stress, perturbed complex IV activity, and apoptosis. Alleviation of oxidative stress using Vitamin E treatment rescued cells from vulnerability to apoptosis.

Cerebellar syndrome was modelled by differentiating patient-derived iPSC into motor neurons.^44^ Mutant CHCHD10 (c.176C > T) caused abnormal mitochondrial morphology, increased sensitivity to staurosporine, and increased glutamate-induced caspase activation. In addition, Pacitti and Bax^45^ modelled MNGIE using iPSC-derived cerebral organoids, successfully recapitulating the absence of thymidine phosphorylase in the various cell types. Finally, Cooper et al.^47^ modelled ATAD3A defect (c.1064G >A) using patient-derived iPSCs differentiated into neurons. The ATAD3A defect led to altered mitochondrial network and increased number of lysosomes, which suggests that neurological phenotypes seen in patients are caused by upregulated basal autophagy and unbalanced mitochondrial dynamics.

### Modelling diseases arising from mutations in mtDNA

Twenty-three papers modelling diseases arising from mutations in mtDNA were identified and are summarised in *Table 3*. Of these, 10 studied MELAS syndrome, one of the most common mitochondrial diseases. Within these papers, 9 were modelling the m.3243A > G mutation in the MT-TL1 gene which is reported to be responsible for approximately 80% of MELAS cases.^72^ Only 4 groups modelled m.3243A > G using patient-derived iPSCs. Neuronal cell types studied included glutamatergic cortical neurons,^20,51,52^ motor neurons and spinal cord organoids^53^ (*Table 2*). Common disease phenotypes observed in these models included impairment of neuronal differentiation and decreased survival of mature neurons, mitochondrial dysfunction, defects in neurite outgrowth and synapse formation, and neuronal network abnormalities.

**Table 3.** Papers modelling mtDNA mutations. Abbreviations: induced pluripotent stem cells (iPSCs), neuronal progenitor cells (NPCs, OXPHOS complex I/IV (CI/CIV), reactive oxygen species (ROS), mitochondrial membrane potential (MTMP), brain-blood-barrier (BBB).

Reference	Gene&Mutation	Model	Key Findings
**MELAS Syndrome**			
^50^	MT-TL1 m.3243A > G	Patient fibroblasts converted directly to iNeurons co-cultured with rat astrocytes	Mutant iNeuronss exhibited impaired mitochondrial respiration, mitochondrial membrane abnormalities, and reduced spontaneous activity.
^51^	MT-TL1 m.3243A > G	Patient-derived iPSCs differentiated to excitatory cortical neurons	High heteroplasmic neurons showed altered neuronal network activity and changes in expression of genes involved in mitochondrial and synaptic function, which improved on Sonlicromanol.
^52^	MT-TL1 m.3243A > G	Patient-derived iPSCs differentiated to excitatory cortical neurons	Mutant neurons with high heteroplasmy displayed impaired mitochondrial function, simpler dendrite structure, less synapses and a distinct neuronal network phenotypes.
^53^	MT-TL1 m.3243A > G	Patient-derived iPSCs differentiated to motor neurons and spinal cord organoids	Elevated Notch signalling impaired differentiation to motor neurons and interfered with neurite outgrowth, possibly via CI inhibition.
^54^	MT-TL1 m.3243A > G	Cybrids from enucleated patient fibroblasts and mtDNA depleted SH-SY5Y cells	Mutant cybrids exhibited impaired CI. Culturing in low glucose resulted in increased respiratory chain subunits and mtDNA copy number. 5-aminoimidazole-4-carboxamide riboside increased levels of CI but also of assembly intermediates.
^55^	MT-TL1 m.3243A > G	Cybrids from enucleated patient fibroblasts and mtDNA depleted SH-SY5Y cells	Mutant cybrids showed impaired CI assembly and respiration, a shift to glycolysis. Ketone bodies/low glucose improved CI function, ATP synthesis, mtDNA level.
^56^	MT-TW m.5541C > T	Patient myoblasts converted to iPSCs and differentiated to NPCs and neurons	Less fully differentiated mutant neurons, but normal number of NPCs.
^20^	MT-TL1 m.3243A > G	Patient-derived iPSCs differentiated to cortical neurons	Mutant neurons showed mtDNA transcriptional changes, reduced CI, defective autophagy, and failed to increase mtDNA copy number after differentiation.
^57^	MT-TL1 m.3243A > G	Modelling BBB, comprising immortalised brain capillary endothelial cells and foetal astrocytes repopulated with 97% mutant mtDNA	Reduced CI and CIV in mutant endothelial cells and astrocytes. Trans endothelial electrical resistance reduced in mutant endothelial cells, indicating a disrupted BBB.
^58^	MT-TL1 m.3243A > G	Olfactory neurospheres derived from a patient	Mutant cells showed reduced MTMP and ATP synthesis and elevated SOX and lactate; treatment with galactose led to improvements.
**LHON**			
^59^	(1) MT-ND1 m.3460G > A (2) MT-ND4 m.11778G > A	Patient-derived iPSCs differentiated to neurons	Mutant neurons showed elevated levels of mitophagy and autophagy and increased likelihood of apoptosis. Idebenone reduced autophagy and apoptosis.
^60^	MT-ND4 m.11778G > A	Cybrids generated using patient platelets and mtDNA depleted SH-SY5Y cells	Treatment with rapamycin led to mTOR inhibition, increased mitophagy, and a gradual decline in heteroplasmy, and ATP levels were rectified, to a degree. Treated mutant cybrid mtDNA copy number and cytotoxicity were not significantly different to controls
^61^	MT-ND4 m.11778G > A	iPSCs converted to NPCs	NPCs were depleted of endogenous mtDNA and repopulated with mutant mtDNA complexed with MTD-TFAM. Introduction of pathogenic mtDNA did not disrupt their differentiation potential.
^62^	MT-ND4 m.11778G > A	Cybrids generated using patient platelets and mtDNA depleted SH-SY5Y cells	Developed a modified TFAM able to enter the mitochondrial compartment in cybrids. MTD-TFAM led to temporary improvement in basal respiration, nuclear encoded CI subunits and mitochondrial mass.
^63^	(1) MT-ND4 m.11778G > A (2) MT-ND1 m.3460G > A	Cybrids from enucleated patient lympho-blasts and mtDNA depleted NT2 cells	mtDNA copy number was reduced after neuronal differentiation in mutant NT2 cells. They also showed increased ROS generation, which was prevented by rotenone.
**MERRF**			
^64^	MT-TK m.8344A > G	Patient fibroblasts converted directly to iNeurons	CoQ10 supplementation improved bioenergetic parameters, spare respiratory capacity and maximal respiration.
^65^	MT-TK m.8344A > G	Patient-derived iPSCs differentiated to NPCs	Mutant NPCs showed elevated expression of antioxidant genes, elevated ROS levels, and mitochondrial fragmentation.
^66^	MT-TK m.8344A > G	Patient-derived iPSCs differentiated to inner ear hair cell -like cells.	Proposed a transcription-factor mediated differentiation into inner ear hair cells. Mutants exhibited elevated ROS levels and fewer stereociliary bundle-like protrusions.
**Leigh Syndrome**			
^67^	MT-ND5 m.13513G > A	Patient-derived iPSCs differentiated to neurons	Mutant NPCs were less likely to cluster and had increased neuronal cell death when co-cultured with mouse astrocytes. Neurons had impaired CI, oxidative phosphorylation and calcium buffering.
^68^	MT-ATP6 m.8993T > G	Patient-derived iPSCs differentiated to neurons	Increased mTORC1 signalling in mutant neurons. Rapamycin improved ATP levels, decreased abnormal AMPK activity, protected against glutamate toxicity.
**Other**			
^69^	MT-ATP6 m.9154C > T	Patient-derived iPSCs differentiated to spinal motor neurons	Mutant NPCs showed impaired ATP synthase assembly and abnormal cristae. Spinal motor neurons exhibited altered metabolism, neurogenesis, and reprogramming, which was depended on the level of heteroplasmy.
^70^	MT-ATP6 m.9185T > C	Patient-derived iPSCs differentiated to NPCs	Homoplasmic mutant NPCs exhibited elevated mitochondrial membrane potential, impaired ATP synthesis and calcium homeostasis, which improved on Avanafil.
^71^	Mt 12S rRNA m.1555A > G; TRMU c.28G > T	Patient lymphoblastoid cells converted to iPSCs and differentiated to inner ear hair cell -like cells	Mutant inner ear hair cells with both m.1555A > G and TRMU variants had more electrophysiological and structural abnormalities than those with m.1555A > G only. They also showed transcriptomic changes relating to mechanotransduction.

Two studies involved the generation of cybrids, in both cases from enucleated patient fibroblasts and mtDNA depleted SH-SY5Y cells.^54,55^ One study modelled the blood-brain barrier (BBB) using immortalised brain capillary endothelial cells and foetal astrocytes with endogenous mitochondria replaced by those with the m.3243A > G mutation at 97% heteroplasmy.^57^ One paper modelled the m.5541C > T mutation, which represents another possible cause of MELAS and impinges on the mitochondrial tRNA tryptophan gene (MT-TW). For this study, authors used patient-derived myoblasts converted into iPSCs which were further differentiated into neuronal precursors cells and neurons.^56^ Common findings in these models include complex I and IV dysfunction, mtDNA copy number changes, substantial loss of differentiated mutant neurons and disrupted BBB integrity. Finally, olfactory neurospheres from a patient with the m3243A > G mutation presented biochemical and metabolic abnormalities, as seen by hyperspectral imaging.^58^

Five papers studied neuronal models of LHON caused by either the m.11778G > A or m.3460G > A mutation in the MT-ND4 gene. Two studies used patient fibroblasts converted into iPSCs and differentiated into neurons or neuronal progenitor cells.^59,61^ An earlier paper used cybrids generated by enucleated patient lymphoblasts and mtDNA depleted NT2 cells to model the disease.^63^ Key pathological changes included elevated levels of mitophagy, autophagy, reactive oxygen species (ROS), and apoptosis. Treating LHON-affected cells with idebenone resulted in a reduction of autophagy, apoptosis, and ROS production.^59^ Moreover, cybrids generated using patient platelets and mtDNA depleted SH-SY5Y cells showed positive changes using rapamycin or specific mitochondrial transcription factors.^60,62^

Three papers studied myoclonic epilepsy with ragged-red fibres (MERRF) caused by the m.8344A > G mutation in the MT-TK gene. In these studies, patient fibroblasts were either directly converted into induced neurons (iNs),^64^ or reprogrammed into iPSCs and then differentiated into neuronal progenitor cells.^65^ Interestingly, one study used patient-derived iPSCs differentiated into inner ear hair cell -like cells.^66^ These models identified elevated ROS levels in mutant cells and showed that Guttaquinon CoQ10 supplementation can lead to bioenergetic improvements.

Two papers studied Leigh syndrome, a common neurological manifestation of PMD, affecting primarily the neurons in the bilateral basal ganglia and the brainstem.^73^. One study modelled the m.13513G > A mutation in the MT-ND5 gene,^67^ and the other the m.8993T > G mutation in the MT-ATP6 gene.^68^ Both papers used patient-derived iPSCs differentiated into neurons. MT-ND5 mutant cells showed impaired differentiation, neural stem cell clustering, and dysfunction in neuronal respiration and calcium buffering. On the other hand, neurons with the MT-ATP6 mutation displayed increased mTORC1 signalling, and improvements in ATP levels were seen upon treatment with rapamycin. Two additional studies modelled PMDs arising from MT-ATP6 mutations. One study demonstrated that m.9154C > T mutant iPSC-derived spinal motor neurons show several abnormalities in metabolism, neurogenesis and reprogramming, which are depended on the level of heteroplasmy.^69^ Moreover, NPCs carrying the m.9185T > C mutation showed impaired mitochondrial function and calcium homeostasis, which improved with avanafil.^70^

Finally, Chen and Guan^71^ identified that iPSC-derived inner ear hair cells bearing both the m.1555A > G mutation in the mitochondrial 12S rRNA gene and the heterozygous c.28G > T mutation in TRMU, exhibit more severe phenotypes in a model of sensorineural hearing loss, suggesting a dual effect of the variants in the mtDNA and the nucleus.

## Discussion

The use of *in vitro* models for disease modelling has emerged as a paramount application in current research practices. Thus far, the investigation of human brain disease has heavily relied on animal models, which, while sharing certain disease-responding mechanisms due to their evolutionary closeness to humans, lack natural manifestations of the pathology. The advent of human-specific *in vitro* models holds promise in mitigating interspecies disparities, potentially decreasing the dependence on animal facilities and expediting drug screening efforts.

Modelling primary mitochondrial diseases is challenging due to the inherent clinical heterogeneity and tissue-specific manifestations. Moreover, the complexity of modelling mitochondrial diseases is compounded by the difficulty in manipulating mtDNA in order to introduce disease-relevant mutations. To effectively delineate the mechanisms driving the phenotypes observed in patients, we require thoroughly characterized models that can recapitulate the underlying metabolic dysfunctions while offering further insights into the molecular pathways involved. The development of reliable neuronal models is of particular relevance as neurological symptoms are prevalent in PMD patients and accessibility to brain tissue is difficult and most often limited to post-mortem samples. While we have been conducting the search described here, one relevant systematic review has been published by Tolle et al.^74^ describing the current state of mitochondrial DNA disease modelling with a focus on mtDNA editing. In addition, a recent review by Harvey et al.^75^ also discussed PMD models, but it was restricted to the modelling of inherited optic neuropathies using iPSCs.

Several different neuronal cellular models have been used to study disease mechanisms and treatments in PMD, each presenting with advantages and limitations. Selecting the most appropriate in vitro model is crucial to reach meaningful conclusions. The main models currently used are i) genetically modified neuroblastoma cell lines; ii) different types of iPSC-derived 2D neurons and iii) 3D cortical organoids generated from patient-derived iPSCs. Neuroblastoma cell lines, such as SH-SY5Y, are widely used to model neurological disorders including PMD due to their easy maintenance and capacity for high-throughput screening. For example, inhibiting CoQ10 successfully triggered a mitochondrial defect in SH-SY5Y cells showing unique consequences of CoQ10 deficiency in neuronal cell types compared to fibroblasts.^49^ While neuroblastoma cell lines can be valuable tools to investigate tissue-specific mechanisms, they are often used as cybrids when modelling mtDNA mutations which can inadvertently undermine relevant interactions between the nuclear and mitochondrial genomes.

Advancements in stem cell technologies during the past decade have provided researchers with an unparalleled advantage in disease modelling, capitalizing on patient-derived cells. Reprogramming readily available somatic cells, such as skin fibroblasts, into induced pluripotent stem cells (iPSCs) provides a cellular model that is easily accessible and has the potential to differentiate into almost any cell type while conserving the relevant genetic background. The majority of the studies included in this review featured iPSC-derived neuronal models, which offer an advantage in modelling PMD as they allow to investigate disease mechanisms in a patient-specific context. Moreover, while brain involvement is common in PMD patients, not all brain regions or cell types are equally affected. Through the use of iPSC-derived models, it will be possible to investigate what may be driving the selective vulnerability of specific neuronal populations. For example, iPSC-derived motor neurons carrying mutations associated with peripheral neuropathy were found to have impaired mitochondrial transport together with altered mitochondrial morphology and distribution.^32,34,35^

While the development of somatic reprogramming technologies opened new exciting avenues for disease modelling, the phenotypic heterogeneity observed in iPSC populations derived from the same donor remains a challenge. Some of this variability has been described to arise from residual DNA methylation and chromatin accessibility signatures from the somatic cells of origin.^76,77^ Moreover, Wei et al.,^78^ showed that somatic reprogramming remodelled the mtDNA landscape in a clone-specific manner, with some mtDNA variants undergoing purifying selection and others being enriched. After reprogramming, iPSCs should be characterized for shifts in heteroplasmy and possible emergence of pathogenic mtDNA mutations. Considering the variability introduced during reprogramming and the spectrum of clinical phenotypes that can result from a single PMD-causing mutation, it is preferable to use multiple iPSC clones from the same patient when investigating disease mechanisms to ensure reproducibility.

More recently, iPSC-derived neuronal differentiation protocols have been adapted for the generation of 3D cerebral organoids. The resulting cellular models recapitulate the microenvironment of the early nervous system and mimic various characteristics of cortical development, with regions of progenitor cells giving rise to several mature neuronal cell subtypes, including supporting glial cells and astrocytes.^79^ For this reason, cerebral organoids are particularly valuable in PMD research since mitochondrial remodelling directly affects neuronal differentiation, which relies on a metabolic shift from glycolysis to OXPHOS. Cerebral organoids allow researchers to investigate mitochondrial dysfunction, its progression during development and how it impacts neuronal differentiation. For example, in models of Leigh syndrome, failure to engage in the metabolic switch to OXPHOS resulted in impaired development of neurons and triggered a shift to astrocytes during cortical development.^29^ Similarly, SURF1 and NDUSF4 mutations restricted differentiation of proliferating cells and hindered neuronal maturation.^30^

Of note, organoid models are technically challenging and limited by the lack of vascularisation, which restricts their growth potential. Organoids are grown on an orbital shaker to facilitate the diffusion of oxygen and nutrients, but once they reach a certain size they can become necrotic. New technologies have been developed to enable longer survival and to overcome the challenges related to poor oxygen supply, such as growing organoid slices at the air-liquid interface (ALI-COS).^80^ ALI-COS can be cultured for over 200 days and exhibit neural networks with functional connectivity.^81^ Current protocols for brain organoids differentiation also lack immune components. However, recent studies have shown that it is possible to incorporate microglia-like cells into brain organoids and culture them for downstream functional studies.^82,83^

The timing of neuronal development is species-specific. In humans, the protracted neuronal maturation is thought to enable the increased complexity and plasticity observed in the human brain. All iPSC-derived neuronal models described in this review were grown for just a few weeks, with 8 weeks being the longest timepoint.^23^ Brain organoids were grown for longer than 2D neurons, with some being cultured for up to 90 days, though this timepoint only corresponds to approximately week 16 post-conception.^30,79^ However, brain organoids in other neurodegenerative diseases have been cultured for over 200 days.^80^ The immaturity of the available neuronal models has to be carefully considered when designing experiments. These models would be better suited to investigate mitochondrial diseases that are thought to affect neuronal development. Of note, it was recently shown that the timing of neuronal development is tightly linked to mitochondrial metabolism. Enhancing mitochondrial oxidative metabolism led to faster neuronal maturation.^84^^,^^85^ While accelerating neuronal maturation might help overcome some of the limitations arising from the immaturity of the models, PMD researchers will need to exert extra caution when attempting to alter the cellular metabolism for this purpose.

The use of in vitro neuronal models is particularly relevant in diseases where animal models are lacking or are not representative of the human disease or the biological process, which is the case for multiple PMDs.^86^ However, it is important to note that while powerful, iPSC models face limitations. The reprogramming process and differentiation itself may introduce variability. Furthermore, patient-derived models lack isogenic controls without subsequent gene editing, which can be cumbersome. Moreover, the maturity of the derived cell types may differ across cell lines and may not recapitulate adult human cells. For these reasons, thorough characterization and validation of the models will be necessary to draw meaningful conclusions.

Future research will continue to benefit from these in vitro models. However, to draw meaningful mechanistic conclusions it will be necessary to integrate new and exciting neuroscience techniques and discoveries into the experimental designs. Most papers in this review, independent of the mutation being modelled, had almost exclusively metabolic readouts. Understanding how the mutations affect mitochondrial dynamics, mitophagy, calcium homeostasis and OXPHOS complexes will identify important insights about the role of these molecular mechanisms in neuronal dysfunction. Interestingly, all the studies that did investigate neuronal pathology reported impaired neuronal differentiation and maturation. Neuronal development is intrinsically linked to timely metabolic shifts.^87^ Further investigation on how specific PMD-related mutations hinder development in specific neuronal cell types could provide valuable insights on tissue-specificity and offer novel avenues for targeted therapies.
